# 3D texture analysis for classification of second harmonic generation images of human ovarian cancer

**DOI:** 10.1038/srep35734

**Published:** 2016-10-21

**Authors:** Bruce Wen, Kirby R. Campbell, Karissa Tilbury, Oleg Nadiarnykh, Molly A. Brewer, Manish Patankar, Vikas Singh, Kevin. W. Eliceiri, Paul J. Campagnola

**Affiliations:** 1 Department of Medical Physics, University of Wisconsin- Madison, Madison, WI 53706, USA; 2 Morgridge Institute for Research, Madison, WI 53715, USA; 3 Department of Biomedical Engineering, University of Wisconsin-Madison, Madison, WI 53706, USA; 4 VU Medical Center, VU University Amsterdam, Amsterdam, Netherlands; 5 Department of Obstetrics and Gynecology, University of Connecticut Health Center, Farmington, CT 06030, USA; 6 Department of Obstetrics and Gynecology, University of Wisconsin-Madison, Madison, WI 53706, USA; 7 Department of Biostatistics and Medical Informatics, University of Wisconsin-Madison, Madison, WI 53706, USA.

## Abstract

Remodeling of the collagen architecture in the extracellular matrix (ECM) has been implicated in ovarian cancer. To quantify these alterations we implemented a form of 3D texture analysis to delineate the fibrillar morphology observed in 3D Second Harmonic Generation (SHG) microscopy image data of normal (1) and high risk (2) ovarian stroma, benign ovarian tumors (3), low grade (4) and high grade (5) serous tumors, and endometrioid tumors (6). We developed a tailored set of 3D filters which extract textural features in the 3D image sets to build (or learn) statistical models of each tissue class. By applying k-nearest neighbor classification using these learned models, we achieved 83–91% accuracies for the six classes. The 3D method outperformed the analogous 2D classification on the same tissues, where we suggest this is due the increased information content. This classification based on ECM structural changes will complement conventional classification based on genetic profiles and can serve as an additional biomarker. Moreover, the texture analysis algorithm is quite general, as it does not rely on single morphological metrics such as fiber alignment, length, and width but their combined convolution with a customizable basis set.

Ovarian cancer accounts for 5% of cancer deaths among women and is the most deadly gynecologic cancer^1^. In 2015, an estimated 21,290 new cases and 14,180 deaths from ovarian cancer are expected in the US. Current screening and imaging techniques are insufficient for early detection as the majority of cases (61%) are diagnosed with widespread metastatic disease, for which the 5-year survival rate is 27%^1^. CA125 is currently the best serum biomarker, however it lacks needed sensitivity and specificity^2^,^3^. A CA125 screen combined with transvaginal ultrasound is provided for women who are at high risk, however, the resulting sensitivity and specificity of this combined approach is still insufficient for screening early tumor growth^4^. With current diagnostic imaging techniques including computed tomography, positron emission tomography, ultrasound and magnetic resonance imaging^5^,^6^,^7^,^8^,^9^, only 15% of cases are diagnosed while localized to the ovary (stage 1) for which 5-year survival is 92%^1^. With these current limitations in screening and imaging modalities^2^,^5^, there remains a compelling need for new technologies that can image early ovarian cancers with better resolution and specificity to improve the accuracy of diagnosis and prognosis and provide new insight into the disease etiology.

Recent studies have shown that ovarian carcinomas are not homogeneous and can be broadly divided into two different types by their respective genetic mutations and epidemiological risk factors, delineated as type I and II^10^,^11^. The former grouping includes low-grade, borderline, and endometrioid tumors, whereas the latter refers to high grade serous carcinomas, which are the most prevalent (~70%) and have the poorest 5-year survival rates (~27%)^1^. This dualistic classification is primarily based on genetics, but as most epithelial cancers have associated extracellular matrix (ECM) remodeling, it is also important to classify the respective alterations in the tumors as this will provide further diagnostic/prognostic information. Probing changes in the ECM, primarily cellular characteristics and collagen architecture requires microscopic resolution not achievable by conventional clinical modalities. While microscopic analysis by H&E histology remains the gold standard for pathologic analysis, it is limited in terms of numbers of sections and, more importantly, is not highly sensitive to collagen fiber organization. A more complete classification based on ECM features could improve the current standards of care.

This is an important consideration as recent studies have demonstrated that there is a close correlation between cancer stages with remodeling of the ECM in the tumor microenvironment (TME) in several carcinomas^12^,^13^,^14^,^15^,^16^. For example, using the collagen specific modality of Second Harmonic Generation (SHG) microscopy, Keely and co-workers identified tumor associated characteristic signatures (“TACS”) of collagen alignment in breast cancer^17^,^18^. We previously used SHG to characterize structural aspects in normal and high grade serous (HGS) ovarian cancer using an integrated approach combining SHG creation physics and optical scattering properties^19^.

While successful, this approach is both experimentally and computationally very intensive. Other reports have analyzed histologic ovarian cancer sections using techniques including whole image Fast Fourier Transforms (FFTs) and grey scale co-occurrence localization matrix (GSCLM)^20^,^21^. However, such conventional analysis approaches are limited in their application to ovarian cancers. This is because, while altered from normal tissues, ovarian cancers do not display the easily identifiable TACS observed in breast cancer. Moreover, as we will demonstrate, the modifications are substantially different for type I (and within the type I group) and type II tumors. Thus, a general and more versatile approach is needed to classify collagen architecture alterations across the spectrum of ovarian cancers. For example, our attempts at classification using FFTs or even 2D wavelet transforms did not achieve sufficient accuracy (<70%) in this setting. High classification accuracy of ovarian cancers is an important goal for classification of given the heterogeneity of tumor types.

Our approach to this problem is to implement a computer vision scheme based on texture analysis by creating “topic models” for each type of ovarian cancer, benign tumors, and normal tissues. In computer vision, texture analysis attempts to quantify overall perceptual qualities (e.g., rough or smooth, or alignment), and repetitive patterns as a function of the spatial variations in pixel intensities around small individual regions in the image. In previous work, we had successfully implemented 2D texture analysis for SHG images using “textons” with k-means clustering and nearest neighbor classification^22^. Textons are repeating features determined by convolution with a basis comprised of a series of edge, bar, Gaussian and Laplacian filters^23^, where we previously used the MR8 basis set. Models for training and testing sets were created and HGS and normal stroma were classified with accuracy>95%^22^. Importantly, this approach is general as it does not rely on direct visuals such as fiber alignment and size.

While successful, we utilized this approach on a series of 2D optical sections of normal and HGS tumors. As SHG has intrinsic 3D imaging capabilities, and the stroma has 3D collagen fiber architecture we are likely missing valuable information. Although 3D texture analysis has been used in radiomics to extract CT/MRI image features to classify tumors^24^,^25^,^26^, to the best of our knowledge, there are no analogous reports using 3D renderings of SHG image data. This represents an un-exploited opportunity in SHG and other nonlinear microscopies. This is because, while such modalities collect 3D data, analysis of 2D optical sections is the most common paradigm.

In this paper, we extend the texton based analysis to 3D by creating tailored 3D versions based on the 2D MR8 filter bank. This is important as the needed filter design in the 3D regime is more complex as the heterogeneity, while yet richer in terms of the texture is far greater than for 2D image data. We further extend the process to analyzing six different ovarian tissues types across a spectrum of disease state, further increasing the heterogeneity of the problem and need for 3D analysis to capture all the texture features. We successfully classified the tissues using a *one vs the rest* approach with better than 83–91% accuracy. By comparison, much lower accuracy, and unsatisfactory performance was achieved using our previous 2D approach when applied to the same six classes.

## Material and Methods

### Tissue acquisition

We conducted an institutional review board (IRB) approved study (University of Connecticut Health Center IRB and University of Wisconsin-Madison School of Medicine and Public Health IRB) of de-identified tissues where the methods were carried out in accordance with the relevant study guidelines. We imaged *ex vivo* ovarian tissues from 5 normal patients, 5 patients with high-grade serous (HGS) ovarian cancer, 5 patients with benign tumors, 5 high risk patients with BRCAI or BRCAII gene mutations or family history, 3 patients with low-grade serous (LGS) tumors, and 5 patients with endometrioid tumors from the University of Connecticut Health Center and University of Wisconsin-Madison. Informed consent was obtained in all cases. The diagnoses for all tissues were confirmed by pathological analysis. For SHG imaging, tissues were fixed in 4% formalin for 24 h, transferred to phosphate buffered saline, and sliced into 100 to 200 μm thick sections using a Leica Vibratome 1200S (Leica Biosystems, Buffalo Grove, IL).

### SHG Imaging

Tissues were imaged by the SHG microscopy as previously described^19^,^27^. The excitation used 890 nm, 100 fs pulses from a Ti:sapphire oscillator (Mira, Coherent, Santa Clara, California). The SHG laser scanning microscope was a modified Fluoview300 (Olympus, Center Valley, Pennsylvania) mounted on a fixed stage upright stand (Olympus BX61). All imaging was performed with a 40× (0.8 NA) water immersion objective lens with an average power of 20 to 50 mW at the focal plane. To excite all orientations equally, circularly polarized light was used throughout. This was achieved at the focal plane using the combination of a quarter wave plate and a half wave plate as a compensator. The SHG was collected in the forward direction by a 0.9 NA condenser, isolated with a 20 nm bandwidth 445 nm bandpass filter (Semrock, Rochester, New York) and detected by a single photon counting photomultiplier tube module (Hamamatsu 7421, Hamamatsu City, Japan). Images were acquired at three times zoom with a field-of-view of 170 μm by 170 μm and a field size of 512 by 512 pixels to sample at the Nyquist frequency. Similarly, axial sectioning was acquired in one micron steps. Considering the reduction of the signal with increasing depth into the tissue, we only employed the top 30–40 μm of the tissue stack to maintain approximately the same level of contrast between images. 3D reconstructions were performed in Bitplane Imaris (Bitplane AG, Zurich, Switzerland).

### Texture Analysis Method

We previously applied 2D texton analysis utilizing k-means clustering to train 2D texture features and adapted nearest neighbor classification to classify normal and HGS ovarian cancer tissues^22^. Here we further develop and adapt this algorithm for 3D texture analysis targeting classification with six different types of ovarian tissues.

**Figure 1:**
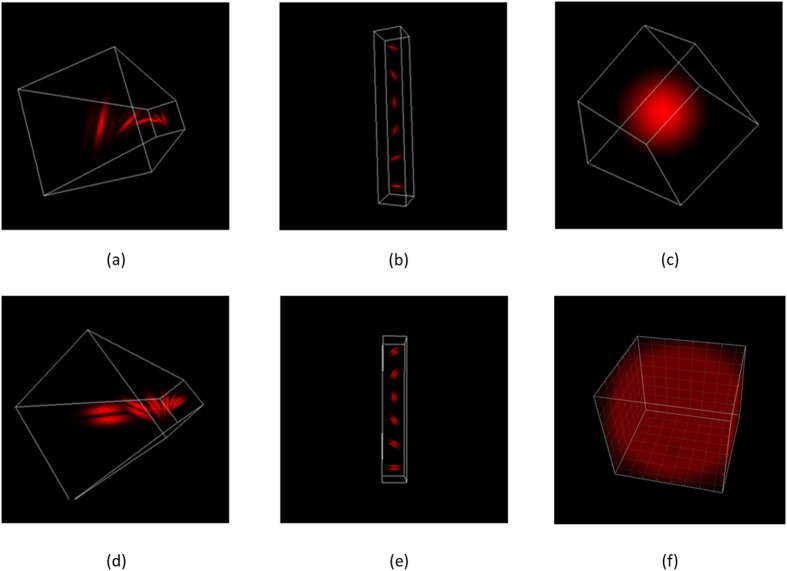
Representative 3D image filters: 6 type-I filters at mid-scale around z-axis (**a**), 6 type-I filters at mid-scale around y-axis (**b**), 3D Gaussian filter (**c**), 6 type-II filters at mid-scale around z-axis (**d**), 6 type-II filters at mid-scale around x-axis (**e**), 3DLaplacian filter (**f**).

We first redesigned the texture filter set to generate a bar/edge like filter to simulate collagen fiber morphology in different directions and different sizes in 3D. We initially wanted to implement the new filter set in all directions and evenly distributed in space which would have been comprised of 6 × 6 × 6 × 3(scales) x 2(types) +2 = 1298 filters, however this was not computationally realistic for the convolution. Therefore, we only chose filters around the x, y, and z axes. The selected 3D filter set is a multi-scale, multi orientation filter bank with 110 filters and is demonstrated in Fig. 1. It consists of first (Fig. 1(d,e)) and second (Fig. 1(a,b)) derivatives of 3D-Gaussians at 6 orientations about x, y and z-axis with three scales, 1 Laplacian of Gaussian (LOG) filters, and 1 Gaussians filter. The Gaussian (Fig. 1(c)) and Laplacian of Gaussian (Fig. 1(f)) filters are generated with delta = 10 pixels; the first and second derivatives of 3D-Gaussians are generated at 3 different scales with (σ_x,_ σ_y_, σ_z_) = {(1, 1, 3), (2, 2, 6), (4, 4, 12)}. Measuring the maximum response only across orientations at each scale about one axis reduces the number of responses from 110 to 20, which provides rotation invariant behavior along each axis.

**Figure 2:**
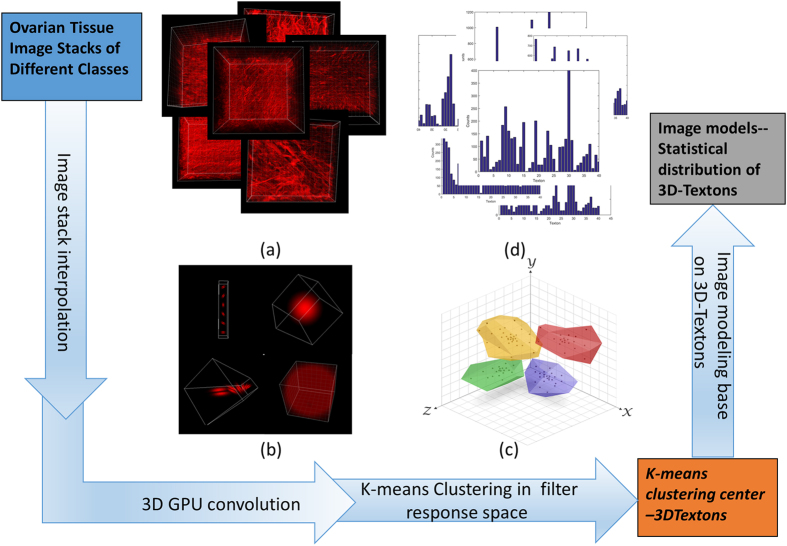
Training stage flowchart (**a**) 3D renderings of SHG 3D images of the six different types of ovarian tissues (**b**) representative 3d filters (**c**) K-means clustering (**d**) histogram models generated from the training images using 40 texton bases.

Secondly, we applied a convolution with this filter bank and the 3D SHG image data. To satisfy the Nyquist criterion, the pixel sizes in the acquired data were 1.0 and 0.37 μm in the axial and lateral directions, respectively. For the convolution, we interpolated the 3D image reconstruction so that the pixel sizes were equivalent. Here we employed GPU computing within the Matlab parallel computing toolbox to greatly decrease the computational time. Further we randomly chose 50 by 50 by 5 voxels in the x, y, z axes from the original 512 by 512 by 60 pixels in the interpolated image stack (180 μm by 180 μm by 20 μm) to keep the computational cost feasible. The chosen voxels filter responses were grouped by k-means clustering which partitions the full set of patch-wise image convolution responses, where each observation belongs to the cluster with the nearest mean, serving as a prototype of the cluster or image feature. Here we clustered the 20 dimensional filter responses from 12,500 randomly chosen voxels into 40 response centers or textons. Similar to the 2D analysis, we found that 40 textons gave the highest accuracy. Then we built a model for each training image stack as a histogram of the determined 3D-texton statistical distribution. The image training workflow is displayed in Fig. 2.

**Figure 3:**
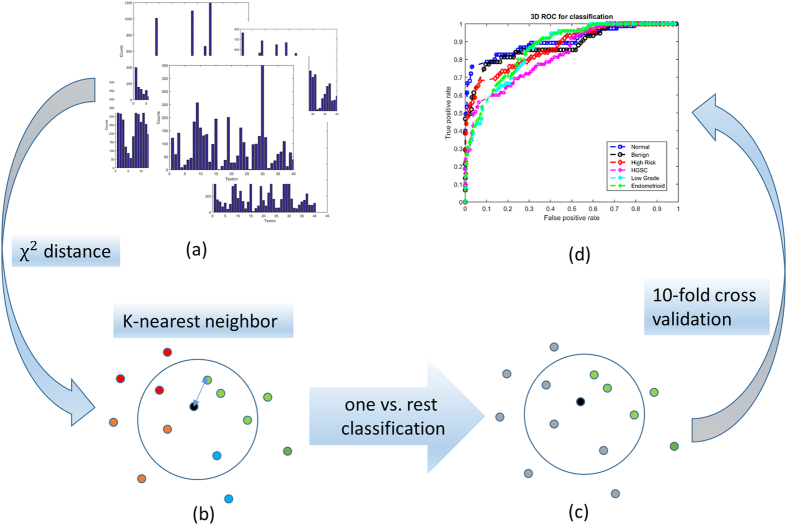
Classification stage flowchart showing (**a**) representative training image models; (**b**) demonstration of the χ2 nearest neighbor (NN) classification with four different classes (circle in four different colors); (**c**) demonstration of one vs. rest classification, where the black circle represent a models of the test image stacks, the green circles represent training models of one of the tissue classes, and the grey circles represent models of the remainder of the classes. (**d**) Resulting ROC curve.

In the classification stage, each testing image was also built into histogram model based on the trained 3D textons. We used the χ^2^ nearest neighbor distance, d, to evaluate differences between the statistical distributions of the textons in the training and testing stacks. Then we performed 10-fold cross validation, where we randomly divided the total number of image stacks into 10 groups. In the cross validation procedure, each group served as the test set once whereas the remaining nine are the training set. This process is then repeated and the accuracy corresponds to the average performance of the model over all folds. Lastly, we performed *one-vs-rest* strategy for multiclass classification. Specifically, the *one-vs-rest* strategy involves training a single classifier per class, with the samples of that class as positive samples and all other image stacks as negatives. This strategy requires the base classifiers to produce a real-valued confidence score for its decision, rather than just a class label. In this case we applied the sum of Gaussian weighting q(exp(−d^2^/σ^2^)) for all nearest neighbors models relative to the testing 3D image to evaluate thresholds for the ROC curve. We determined σ from fitting a Gaussian distribution to all χ^2^ distances from each training image pairs. Additionally, we added q as the weighting factor (~5–10) for all the positive cases and (−1) for all the negative cases for optimization of the one-vs-rest classification. This weighting accounts for the semi-balanced classification method. Figure 3 demonstrates the workflow for the classification process.

In this study, we adapted 75 image stacks for each of the six types of ovarian tissues. To provide a comparison of the new 3D texture analysis and our previously reported 2D method (now expanded to multiclass classification (6 tissue types)), for the latter we extracted five randomly chosen single optical sections from the 3D stacks to serve as training set images.

## Results

Our 3D texture classification algorithm is utilized for analyzing 3D SHG image data from six different types of ovarian tissues whose class was initially identified by pathology: (1) normal ovarian stroma with no evidence of disease; (2) high risk ovarian tissue from patients with BRCAI/II gene mutations or family history but without cancer initiation/progression;(3) benign ovarian tumors lacking the ability to invade neighboring tissue or metastasize; (4) Low grade serous (LGS) carcinomas which often have a non-invasive serous borderline component; (5) endometrioid tumors of the ovary that closely mimic their uterine counterparts; and (6) HGS carcinomas. In the dualistic classification scheme^10^, endometrioid and LGS tumors are classified as type I tumors, and HGS are type II. The latter accounts for 70% of ovarian carcinomas and are usually associated with BRCAI/II and p53 gene mutations.

**Figure 4:**
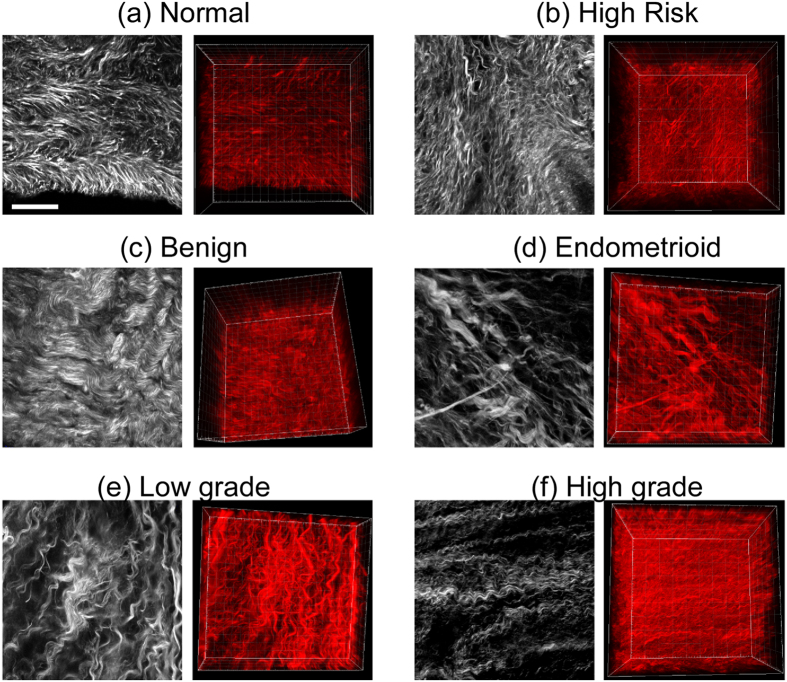
Representative SHG single optical sections (left) and 3d renderings (right) of normal (**a**), high risk (**b**), benign tumor (**c**), endometrioid tumor (**d**), low grade serous (**e**) and high grade (**f**) serous cancer human ovarian tissues. Scale bar = 40 μm.

In Fig. 4, we first present a representative single SHG optical section and 3D rendering from the six different types ovarian tissues, where (a) *ex vivo* normal stroma is characterized by shorter collagen fibers arranged in a mesh-like pattern; (b) high risk tissues are more heterogeneous and have a mixture of curvy and straight fibers; (c) benign tumor tissues are characterized by thicker, short wavy fibers relative to normal; (d) endometrioid tumors also have a high degree of alignment but have sparser fibers than HGS (f), which have characteristic long wavy fibers; and (e) LGS tumors appear fibrotic relative to normal but with shorter fibers than HGS. These overall appearances are common in all the respective tissues and form the basis of using machine learning for classification. However, because of the different typical morphologies, metrics such as fiber size and alignment were not sufficient and we use the 3D texture analysis described in the Methods and the flow charts in Figs 2 and 3.

**Figure 5:**
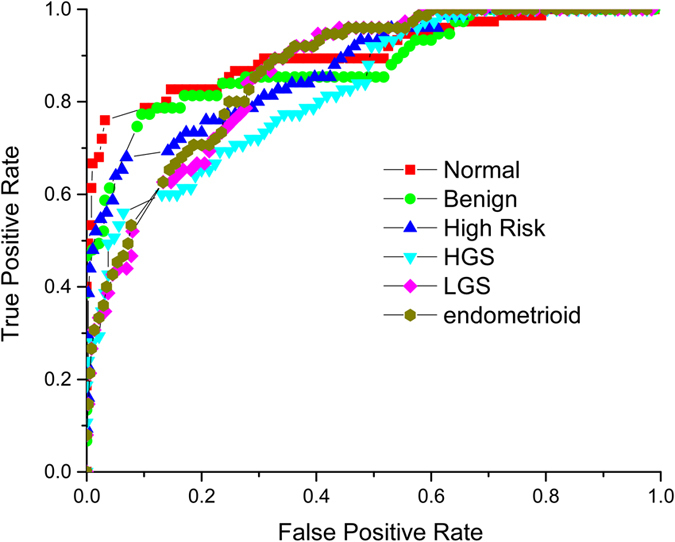
Receiver operating characteristic curves for one vs rest 3D classification of normal ovarian tissue 90.6% accuracy (red squares); benign ovarian tumor 88.3% accuracy (light green circles); high risk ovarian tissue 87.5% (blue triangles); high grade serous cancer 83.0% (turquoise triangles); low grade serous cancer 86.3% (pink diamonds); and endometrioid cancer 86.8% (yellow circles).

To determine the accuracy of classification, we use the receiver operator characteristic (ROC) curves of true positives versus false positives (or sensitivity versus 1-specificity) to determine the accuracy of the classification, where the accuracy is defined as the area under the ROC curve (AUROC). We first optimized the Nearest Neighbor (NN) number, i.e. the number of texton models around the testing sample, where similar to 2D analysis we found NN = 10. Then we systematically changed the texton number to best represent the features in each tissue type. We found 40 textons and NN = 10 to provide the best sensitivity. Using these parameters, we achieved good accuracy with AUROC for the 6 different classes of tissues: normal stroma 90.6%, benign tumors, 88.3%, high risk stroma 87.5%, HGS tumors 83.0%, LGS tumors, 86.3% and endometrioid tumors 86.8% (Fig. 5). For clinical applications, ROC accuracy >80 is required.

**Figure 6:**
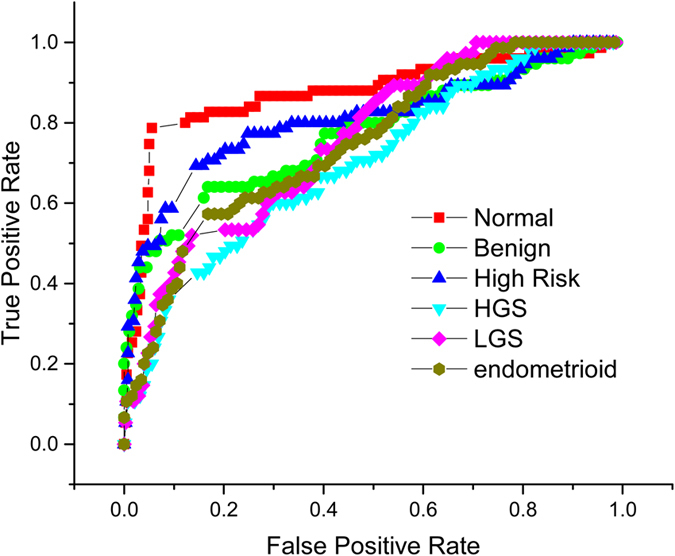
Receiver operating characteristic curves for one vs rest 2D classification of normal ovarian tissue 89.8% accuracy (red squares); benign ovarian tumor 81.0% accuracy (light green circles); high risk ovarian tissue 84.6% (blue triangles); high grade serous cancer 76.5% (turquoise triangles); low grade serous cancer 82.1% (pink diamonds); and endometrioid cancer 76.8% (yellow circles).

To assess the performance of the new 3D implementation, we also employed our previous 2D texture analysis but now included all 6 ovarian tissue classes. Here we built a 2D texture imaging library with five randomly selected single optical sections from the 3D image stack. We found lower accuracies of 89.8% for normal; 81.0% for benign tumors; 84.6% for high risk tissue; 76.5% for HGS, 82.1% for LGS, and 76.8% for endometrioids (Fig. 6). In sum, the 3D texture analysis significantly outperforms the analogous 2D approach for multi-class classification.

## Discussion

### Structural significance

The overall relatively poor performance of standard screening/imaging tools^5^,^28^ suggests that early detection of ovarian cancer demands new technologies that have the resolution and the specificity to detect and monitor small lesions. We have investigated using 3D texture analysis of SHG images of the ECM across the spectrum of ovarian tissues as step in this direction. Instead of extracting visually apparent features like angular distribution, fiber length, or area covered, as has been more commonly done, we convolved 3D image filters with 3D image patches. This is an important distinction, as in real tissues it is often difficult to discretize all of the individual fibers. Moreover, not all ECM modifications have easily apparent changes in fiber architecture, and subtle variations require a sensitive computer vision algorithm for feature extraction. Such analysis can be, however, more difficult by only considering 2D morphology.

We previously performed an analogous 2D analysis where we limited the application to HGS and normal ovarian tissues, which have the largest visual changes in fiber alignment^22^. The task is more difficult for the other tissue classes, where the changes are not as pronounced or have more heterogeneity. For example, in the case of high risk tissues, there is visible heterogeneity not only between patients, but even within the same tissue specimen. By contrast, the respective overall morphologies of HGS tumors and normal stroma are conserved between patients. Comparisons of the 2D vs 3D results are summarized in Table 1, and we found significant improvement with increased dimension, especially for the high risk patients. From a diagnostic/prognostic perspective, this is the most important class, given the high probability of developing HGS cancer in the patient lifetime. The increase in accuracy with 3D vs 2D texture analysis likely arises from two factors. First, the 3D texture analysis includes 3D information from the z axis and provides a more complete picture of the ECM structure. This level of data is most often not exploited in nonlinear optical microscopy, despite the intrinsic optical sectioning capabilities. Second, the 3D stack is a combination of tens of 2D images. Therefore, the image library itself is much more sensitive and tolerant to diversity and heterogeneity of the ECM over the imaged depth profile.

We also note that the overall accuracies achieved here for 2D and 3D are slightly lower than for the 2D HGS vs normal in our previous work. This is a natural consequence of increasing the number of classes from 2–6, and also because those tissues displayed the most substantial and uniform ECM changes. The differences between some of the others classified here, e.g. benign tumors and low grade serous tumors, are subtler in nature. Still, our classification results are consistent with HGS, LGS and endometrioid carcinomas being essentially distinct diseases based on genetic profiles, growth patterns and response to chemotherapy. However, in the dualistic type I and II ovarian cancer classification, LGS and endometrioid tumors are often grouped together^11^, whereas our classification clearly delineates them. This is also consistent with work from our lab using SHG creation physics and optical scattering to characterize the ECM changes in these tissues^19^. Importantly, none of our other metrics were able to differentiate high risk tissues due to the heterogeneity between and within specimens, whereas the 3D texture analysis here was successful in this task.

### Texture analysis considerations

The 3D textons used to build the histogram model for each image stack represent image features described by the whole filter bank. The number of image features is optimized based on that which provides the best representation of the image library. However, as we previously showed, using more textons may not provide higher accuracy as this can over describe the class (in other words, overfit)^22^. On the other hand, too few textons will not provide sufficient discrimination. For these tissues, with our customized filter bank, we have found 40 textons to be optimal. We must also consider the size of the 3D voxel “patches” to convolve with the 3D filter set. Here we randomly selected patches of 50*50*5 out of 512*512*60 voxels for the analysis so that the computational time is reasonable for imaging feature clustering. Then using 40 textons, a 12,500 element filter response vector is needed to construct an effective statistical distribution model of the 3D image set.

To avoid any potential artifacts, we constrain ourselves to using similar SHG intensities in the analysis. Since the SHG intensity rapidly decreases even within 100 microns of stromal thickness, we limit the analysis to voxels within the first 40–50 microns. This is not a major constraint as we have shown that the stroma modifications mostly occur within the first 100–200 microns of thickness, and are most pronounced near the tissue surface^19^.

We use one vs. rest multi-class classification, which is a strategy that involves training a single classifier per class, with the samples of that class as positive samples and all other samples as negatives. This strategy requires the base classifiers to produce a real-valued confidence score for its decision. Although our training set is semi-balanced across all six classes, the one vs. rest binary classification learners result in unbalanced distributions because typically the set of negatives they locate is much larger than the set of positives. Therefore, before decision making, we add a parameter weight (q) and optimize it for each test image to acquire the best accuracy. Also the number of nearest neighbors in the classification algorithm can also be optimized for the accuracy of the ROC curve.

We note that other more sophisticated classifiers such as support vector machines and linear discriminants could also be used. However, their parametrization can be difficult. In contrast, KNN classification is much easier with which to work and operates with fairly simplistic assumptions on the data distribution. Separately, our goal was also to emphasize the value of the texture representations in this application and the KNN classifier itself was sufficient. Thus, most of the high sensitivity/specificity is due to the texture representation scheme rather than the downstream statistical machinery.

### Diagnostic potential

ECM alterations are thought to be a critical step in the intiation and progression of many epithelial carcinomas^12^,^29^,^30^,^31^ and these are increasingly suggested as potential biomarkers^18^,^19^,^20^. In ovarian carcinogenesis and progression, changes in the reactive stroma can occur in the form of increaed collagen concentration (i.e. desmoplasia), increased alignment, and changes in collagen isoform expression^12^. We have previosuly studied these changes across different size scales using a combination of 3D imaging in combination with the measurement of bulk optical properties and Monte-Carlo simulations^19^. These studies revealed differences in collagen sub-resolution fibril and fiber architecture and best differentiate HGS tumors from the other classes, where the classification was moderately successful. While the texture analysis here does not provide the same detailed sub-resolution structural information, it provided equal if not better classification. Importantly, this analysis only requires standard 3D image data, whereas the other studies need directionally resolved SHG data and optical scattering measurements.

Importantly, the required SHG images for the texture analysis could be acquired minimally invasively in conjunction with a standard ovarian laparascope, where backward acquired image data is sufficient. While this could not be used widely a screening tool, it could be applied periodically to high risk patients with known BRCA mutations. Currently the standard of care is to remove suspicious ovaries but this comes with cost of quality of life considerations as well as increased risk factors for other cancers.

## Conclusions

We applied a new 3D texture analysis algorithm to evaluate the ECM structural changes in normal ovarian stroma, high risk ovarian stroma, benign ovarian tumors, and low and high grade ovarian serous cancers, and endometrioid tumors observed by 3D SHG image data. By optimizing the number of textons, testing imaging weighting, nearest neighbor numbers, we achieved high accuracies between ~83–91% between the classes, which greatly outperformed the analogous 2D version. This successful application demonstrates the power of quantitative computer vision evaluation of 3D SHG image features as potential biomarker for cancer stage/type evaluation. Importantly, it does not rely on extracting simple fiber characteristics such as size and alignment. This classification algorithm is a general method based on pre-trained SHG images and is well suited for analysis of rapidly changing fibrillar features for different kinds of tissues.

The hand crated filters used here are not necessarily optimal for image feature extraction for other cancer types or other disease states but can be readily customized for new applications. Also, the effectiveness of the algorithm depends on the diversity of the acquired image library. With the coming era of big data and personalized medicine, our image analysis may assist pathologists in diagnoses and surgeons and oncologists with treatment decisions.

## Additional Information

**How to cite this article**: Wen, B. *et al*. 3D texture analysis for classification of second harmonic generation images of human ovarian cancer. *Sci. Rep.* **6**, 35734; doi: 10.1038/srep35734 (2016).

## Figures and Tables

**Table 1:** The accuracy of classification for 2D and 3D texton analysis with 1 (left column) and 5 (middle) random optical section(s) from the middle axial region of each stack and the corresponding 3D texton analysis.

Accuracy	2D texton 1 section	2D texton 5 sections	3D texton
Normal	87.4%	89.8%	90.6%
Benign	77.1%	81.0%	88.3%
High Risk	80.7%	84.6%	87.5%
High Grade	70.3%	76.5%	83.0%
Low Grade	75.7%	82.1%	86.3%
Endometrioid	75.1%	76.8%	86.8%
